# Supplementary material for: Effect of packaging in preventing cholesterol autoxidation in milk chocolates for a higher quality and safer shelf-life
Source: PLoS One. 2023 Apr 20;18(4):e0284691. doi: 10.1371/journal.pone.0284691 (PMC10118114; doi:10.1371/journal.pone.0284691)
Supplement: Attachment — Submitted filename: Response to Reviewers.docx [file pone.0284691.s001.docx]

Dear Editor,

hereafter please find our point-by-point response to your questions, advises and comments. Thanks for the time you are spending in handling and also revising our manuscript.

Journal Requirements:

When submitting your revision, we need you to address these additional requirements.

1. Please ensure that your manuscript meets PLOS ONE's style requirements, including those for file naming. The PLOS ONE style templates can be found at

https://journals.plos.org/plosone/s/file?id=wjVg/PLOSOne_formatting_sample_main_body.pdf and

<https://journals.plos.org/plosone/s/file?id=ba62/PLOSOne_formatting_sample_title_authors_affiliations.pdf>

*Now the manuscript should meet PLOS ONE's style requirements.*

2. We note that the grant information you provided in the ‘Funding Information’ and ‘Financial Disclosure’ sections do not match.

When you resubmit, please ensure that you provide the correct grant numbers for the awards you received for your study in the ‘Funding Information’ section.

*We will correct the online information, this study does not receive any awards and we do not have a grant numbers, so probably it is not necessary the “Funding statement” but only the “Financial disclosure”.*

3. Thank you for stating the following financial disclosure:

*We have now updated the financial disclosure as follow:*

**Financial disclosure:** This research was funded by Soremartec Italia Srl, Alba (CN), Italy. The funder provided support in the form of salaries for authors Federico Canzoneri, Matteo Arveda, Rosanna Zivoli, Andrea Peraino and Roberto Menta who are employed by Soremartec Italia Srl, Alba (CN, Italy). At the time of conceptualization, Davide Risso was employed by Soremartec Italia Srl, while his current employer is Tate & Lyle Italy SpA. Giuseppe Poli and Valerio Leoni have scientific consultancy contracts with Soremartec Italia Srl.

4. Thank you for stating the following in the Competing Interests section:

*We have now updated the competing interests as follow:*

**Competing interests:** Federico Canzoneri, Matteo Arveda, Rosanna Zivoli, Andrea Peraino and Roberto Menta are employed by Soremartec Italia Srl, Alba (CN, Italy). At the time of conceptualization, Davide Risso was employed by Soremartec Italia Srl, while his current employer is Tate & Lyle Italy SpA. Giuseppe Poli and Valerio Leoni have scientific consultancy contracts with Soremartec Italia Srl. The above mentioned authors were involved in the study design, collection, analysis, interpretation of data, the writing of this article and the decision to submit it for publication. All authors declare no other competing interests. This does not alter our adherence to PLOS ONE policies on sharing data and materials.

***Reviewer #1:***

The authors investigated how long the three prototype milk chocolates could be safely kept on the market, adopting as a quality marker the generation of non-enzymatic cholesterol oxides, expanding the spectrum of non-enzymatic COPs analysis by including the quantification of 7αOHC as well. At the same time, the protective effect of two different food packaging in mitigating the generation of non-enzymatic COPs in the three prototype milk chocolates after 3, 6, 9, 12 months of shelf-life was determined. The results demonstrate a suitable packaging represents an affordable and very promising strategy to prevent the autoxidation reactions in milk chocolate.

This study is well prepared and organized. The results are good and thoroughly discussed. Some major changes are required.

1. In the introduction: The authors should introduce the packaging materials that have been frequently used in packaging milk chocolate.

*This information is already present in the packaging characteristic section, we have now modified the abstract adding some information on the packaging materials used in the study.*

2. In materials and methods: Statistical analysis should be added for data analysis.

*We have now added a paragraph with the statistical analysis.*

3. Packaging characteristics section should test the physical properties (such as WVP, OP, mechanical properties) of the two different packaging materials selected.

*In the packaging characteristics section, we have now added some information about WTR, OTR, VOC*.

4. Data in the tables should be statistically analyzed.

*We have done the statistical analysis and now added the significance in the tables with related comments in the text.*
